# Neutrophil to lymphocyte ratio and platelet to lymphocyte ratio can predict the severity of gallstone pancreatitis

**DOI:** 10.1186/s12876-018-0748-4

**Published:** 2018-01-25

**Authors:** Seung Kook Cho, Saehyun Jung, Kyong Joo Lee, Jae Woo Kim

**Affiliations:** Department of Internal Medicine, Yonsei University Wonju College of Medicine, 20 Ilsan-ro, Wonju-si, 26426 Republic of Korea

**Keywords:** Acute pancreatitis, Gallstone, Neutrophil to lymphocyte ratio, Platelet to lymphocyte ratio, Severity

## Abstract

**Background:**

Neutrophil to lymphocyte ratio (NLR) and platelet to lymphocyte ratio (PLR) predict severity in various diseases. In this study, we evaluated the value of NLR and PLR as prognostic factors in acute pancreatitis (AP).

**Methods:**

Patients with AP were prospectively enrolled from March 2014 to September 2016 at Yonsei University Wonju College of Medicine. NLR and PLR were obtained at admission and were compared with other known prognostic scoring systems.

**Results:**

A total of 243 patients were enrolled with an etiology of gallstone (*n* = 134) or alcohol (*n* = 109). NLR (17.7 ± 18.3 vs. 8.8 ± 8.4, *P* <  0.001) and PLR (344.1 ± 282.6 vs. 177.8 ± 150.1, *P* <  0.001) were significantly higher in the gallstone AP group than in the alcoholic AP group. For gallstone AP, NLR and PLR were significantly higher in severe AP, whereas high NLR and PLR were not related to severe AP in alcoholic AP. For the gallstone AP group, NLR and PLR demonstrated a predictive value significantly superior to C-reactive protein (CRP), whereas NLR, PLR, and CRP were not significant predictors for alcoholic AP.

**Conclusion:**

Our study demonstrated that NLR and PLR can predict the severity of AP, but only in gallstone AP.

## Background

Acute pancreatitis (AP) is an inflammatory process in which local pancreatic injury leads to systemic inflammation through activation of cytokine cascades [1]. The clinical extent of AP varies widely from no symptoms to systemic inflammatory response syndrome (SIRS), persistent organ failure (POF), and death [2]. The clinical presentation of AP is both unreliable and nonspecific and exhibits a sensitivity less than 40% for the prediction of adverse outcomes [3]. Also, the underlying pathophysiology behind the progression of local pancreatic injury to SIRS is not fully understood [4]. Due to the diverse presentations of AP and its unknown pathophysiology, multiple severity scoring systems have been designed to help clinicians in triaging AP patients and predicting their prognosis. The Ranson score, the Acute Physiologic Assessment and Chronic Health Evaluation II (APACHE II) score, the Bedside Index for Severity in Acute Pancreatitis (BISAP) score, and the Glasgow-Imrie criteria are currently in wide use. However, these systems are time-consuming and difficult to apply to patients outside of intensive care settings because they use many variables [5]. Also, they are unsuitable for the evaluation of patients at the time of admission or shortly thereafter. Simplified serum markers such as C-reactive protein (CRP), procalcitonin, interleukin-6, and interleukin-8 have been applied to predict the prognosis or severity of AP, but they are expensive, not readily available, and cannot adequately predict the prognosis or severity of AP [6].

Recently, many research groups have studied the value of hematological components, such as the neutrophil to lymphocyte ratio (NLR) and the platelet to lymphocyte ratio (PLR), in predicting disease severity and outcomes across a variety of diseases, including inflammation, cardiovascular disease, and neoplastic states [2]. The superiority of NLR over total white blood cell (WBC) count, which is used in the Ranson, APACHE-II, and Glasgow-Imrie scoring systems, has been demonstrated in a variety of medical conditions [7]. Furthermore, a few studies have shown that PLR is superior to NLR as a prognostic factor in certain disease conditions [8–10]. Increased NLR and PLR ratios have been associated with inflammatory conditions, and poor outcomes in severe AP are explained by uncontrolled SIRS and its progression to multi-organ dysfunction syndrome [6].

Although a few studies have considered NLR and its prognostic value in AP, no studies have yet examined the prognostic value of PLR in AP. In the present study, we evaluated NLR and PLR values as independent prognostic factors for adverse outcomes in AP and sought to improve previous scoring systems by incorporating NLR and PLR.

## Methods

### Patients

**Fig. 1:**
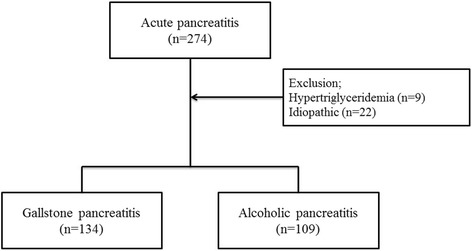
Flow chart of patient enrollment

Patients with AP were prospectively enrolled in Yonsei University Wonju College of Medicine from March 2014 to September 2016. The International Review Board for Human Research of Yonsei University Wonju College of Medicine approved this study (CR315005–002). We only included patients who visited our hospital for a primary visit: patients referred from other clinics were excluded. Written informed consent was obtained from all patients. The diagnosis of AP requires 2 of the following 3 criteria [1]: typical abdominal pain; serum amylase or lipase elevation ≥ 3 times the upper limit of normal; and characteristic findings of AP on contrast-enhanced computed tomography, magnetic resonance imaging, or abdominal ultrasonography. During the study period, a total of 274 patients were diagnosed with AP (Fig. 1). Etiologies other than gallstone and alcohol were excluded. All patients were followed until discharge from the hospital or hospital mortality.

### Data collection

Blood samples for hematological and biochemical data were obtained within 1 h of admission. NLR and PLR were defined as the quotient of absolute neutrophil count to absolute lymphocyte count and that of absolute platelet count to absolute lymphocyte count, respectively. Ranson score, computed tomography scoring index (CTSI), and BISAP score were also calculated upon admission.

### Definition of persistent organ failure

Organ failure was diagnosed as a score ≥ 2 in one or more of the three organ systems described in the modified Marshall score: respiratory failure if the ratio of PaO2/FiO2 was < 300 mmHg; renal failure if serum creatinine was ≥ 1.9 mg/dL; and cardiovascular failure if systolic blood pressure was < 90 mmHg despite fluid replacement. POF was defined as organ failure lasting more than 48 h. That definition is in accordance with the revised Atlanta classification [1].

### Statistical analysis

Continuous variables are presented as mean and standard deviation. Categorical variables are presented as frequency and percentage. Continuous variables in 2 groups were compared using Student’s *t*-test, and categorical variables were compared using the chi-square test. In addition, comparisons of area under the curve (AUC) were used to assess the predictive ability for POF. *P* values less than 0.05 were considered statistically significant, and all statistical analyses were performed using SPSS software, version 18.0 (SPSS Inc., Chicago, IL).

## Results

### Patient characteristics

A total of 243 patients were enrolled (Table 1). The etiologies of acute pancreatitis were gallstone (*n* = 134) and alcohol (*n* = 109). Mean age was higher in the gallstone AP group, whereas the male-to-female ratio and proportion of smokers were lower. We found no significant difference in body mass index or proportion of patients with diabetes mellitus in the two groups. Hypertension and liver cirrhosis were more frequent in patients with alcoholic AP than in those with gallstone AP. According to the Atlanta classification, more patients with moderately severe and severe pancreatitis were classified into the alcoholic AP group. Mean Ranson and BISAP scores on admission did not differ between gallstone and alcoholic AP, but mean CTSI was significantly higher in the gallstone AP group. The mean duration of hospital stay did not differ between the two groups. However, the number of admissions to the intensive care unit (ICU) and mortality were significantly higher in the alcoholic AP group.

**Table 1:** Baseline Characteristics

Characteristics	Overall (*N* = 243)	Gallstone (*N* = 134)	Alcohol (*N* = 109)	*P* value
Age (year)	59.3 ± 17.9	66.3 ± 17.2	50.8 ± 14.8	< 0.001
Sex (male)	166 (68.3%)	72 (53.7%)	94 (86.2%)	< 0.001
Smoking	114 (46.9%)	37 (27.6%)	77 (70.6%)	< 0.001
BMI (kg/m2)	23.8 ± 3.9	24 ± 3.4	23.7 ± 4.4	0.591
Diabetes mellitus	62 (25.5%)	35 (26.1%)	27 (24.8%)	0.810
Hypertension	85 (35%)	56 (41.8%)	29 (26.6%)	0.014
Liver cirrhosis	21 (8.6%)	7 (5.2%)	14 (12.8%)	0.036
Atlanta classification				< 0.001
Mild	161 (66.3%)	107 (79.9%)	54 (49.5%)	
Moderately severe	57 (23.5%)	21 (15.7%)	36 (33%)	
Severe	25 (10.3%)	6 (4.5%)	19 (17.4%)	
Scoring systems				
Ranson	2.4 ± 1.7	2.4 ± 1.5	2.4 ± 1.8	0.998
CTSI	1.9 ± 1.7	1.2 ± 1.3	2.6 ± 1.8	< 0.001
BISAP	1.3 ± 1.1	1.2 ± 1.1	1.4 ± 1.3	0.236
Laboratory data				
WBC (/mm^3^)	11,936 ± 5569	12,023 ± 5172	11,828 ± 6043	0.787
Neutrophil (/mm^3^)	10.2 ± 7.1	10.8 ± 8.1	9.4 ± 5.4	0.126
Lymphocyte (/mm^3^)	1.2 ± 1.0	1.1 ± 1.0	1.5 ± 0.9	< 0.001
Platelet (/mm^3^)	210,000 ± 88,000	220,000 ± 74,000	198,000 ± 102,000	0.001
NLR	13.8 ± 15.3	17.7 ± 18.3	8.8 ± 8.4	< 0.001
PLR	269.5 ± 246.6	344.1 ± 282.6	177.8 ± 150.1	< 0.001
CRP (mg/dL)	4.9 ± 6.9	4.4 ± 5.7	5.5 ± 8.1	0.192
Hospital stay (day)	7.2 ± 6.5	6.9 ± 5.9	7.7 ± 7.2	0.292
ICU admission, n	52 (21.4%)	15 (11.2%)	37 (33.9%)	< 0.001
Mortality, n	15 (6.2%)	3 (2.2%)	12 (11%)	0.005

*BMI* indicates body mass index, *CTSI* computed tomography severity index, *BISAP* Bedside Index for Severity in Acute Pancreatitis, *WBC* white blood count, *NLR* neutrophil to lymphocyte ratio, *PLR* platelet to lymphocyte ratio, *CRP* C-reactive protein, *ICU* intensive care unit

### WBC, neutrophil, lymphocyte, NLR, PLR, and CRP levels on admission

Although the total WBC and neutrophil counts were similar between the two AP groups, the gallstone AP group showed significantly lower lymphocyte count and higher platelet count (Table 1). NLR (17.7 ± 18.3 vs. 8.8 ± 8.4, *P* < 0.001) and PLR (344.1 ± 282.6 vs. 177.8 ± 150.1, *P* < 0.001) were significantly higher in the gallstone AP group than in the alcoholic AP group. CRP did not differ significantly between the groups (4.4 ± 5.7 vs. 5.5 ± 8.1 mg/dL, *P* = 0.192).

We performed subgroup analysis according to AP etiology (Tables 2 and 3). For gallstone AP, NLR was significantly higher in severe pancreatitis, as defined by the revised Atlanta classification (32.4 ± 30.9 vs. 17.1 ± 17.4, *P* = 0.045), Ranson score ≥ 3 (24.8 ± 19.6 vs. 12.1 ± 15.1, *P* < 0.001), and BISAP score ≥ 3 (28.6 ± 20.7 vs. 16.3 ± 17.6, *P* = 0.012) (Table 2). A similar pattern was found for PLR in gallstone AP. In the alcoholic AP group, higher NLR was significantly correlated only with Ranson score ≥ 3 (11.9 ± 109 vs. 6.5 ± 4.7, *P* < 0.001) (Table 3).

**Table 2:** Neutrophil to Lymphocyte Ratio and Platelet to Lymphocyte Ratio in Gallstone Pancreatitis

Parameters	NLR	*P* value	PLR	*P* value
Atlanta classification		0.045		0.008
Mild/moderate	17.1 ± 17.4		330.2 ± 263.7	
Severe	32.4 ± 30.9		641 ± 498.6	
Ranson		< 0.001		< 0.001
< 3	12.1 ± 15.1		258.5 ± 219.3	
≥ 3	24.8 ± 19.6		449.7 ± 316.3	
CTSI		0.083		0.095
< 3	16.3 ± 17.9		323.1 ± 273.7	
≥ 3	23.1 ± 19.1		423.6 ± 306.5	
BISAP		0.012		0.020
< 3	16.3 ± 17.6		323.2 ± 267.6	
≥ 3	28.6 ± 20.7		498.1 ± 347.9	

*CTSI* computed tomography severity index, *BISAP* Bedside Index for Severity in Acute Pancreatitis, *NLR* neutrophil to lymphocyte ratio, *PLR* platelet to lymphocyte ratio

**Table 3:** Neutrophil to lymphocyte ratio and platelet to lymphocyte ratio in alcoholic pancreatitis

Parameters	NLR	*P* value	PLR	*P* value
Atlanta classification		0.606		0.218
Mild/moderate	8.7 ± 8.3		186 ± 150.5	
Severe	9.8 ± 8.8		139.1 ± 145.4	
Ranson		0.001		0.511
< 3	6.5 ± 4.7		169.5 ± 115.4	
≥ 3	11.9 ± 10.9		188.7 ± 187.1	
CTSI		0.107		0.805
< 3	7.5 ± 7.8		181.4 ± 147.4	
≥ 3	10.1 ± 8.7		174.3 ± 153.9	
BISAP		0.417		0.876
< 3	8.5 ± 7.2		179 ± 130.7	
≥ 3	10.1 ± 11.6		173.5 ± 208.2	

*CTSI* computed tomography severity index, *BISAP* Bedside Index for Severity in Acute Pancreatitis, *NLR* neutrophil to lymphocyte ratio, *PLR* platelet to lymphocyte ratio

### Predictive value of NLR and PLR in comparison to CRP in both groups

**Fig. 2:**
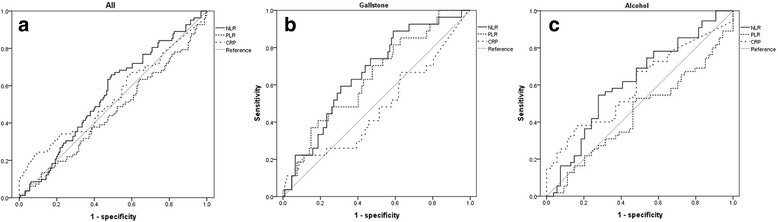
ROC curve to predict persistent organ failure. **a** In all cases, (**b**) In gallstone pancreatitis, (**c**) In alcoholic pancreatitis

We calculated the AUCs of NLR, PLR, and CRP for predicting POF in all patients (Fig. 2a). For all AP patients, NLR, PLR, and CRP failed to predict POF with statistical significance. The AUC was recalculated for the etiology subgroups. For the gallstone AP group, NLR and PLR demonstrated a predictive value significantly superior to that of CRP (NLR-AUC 0.663, 95% CI 0.56–0.77; PLR-AUC 0.638, 95% CI 0.53–0.75; CRP-AUC 0.475, 95% CI 0.35–0.60, Fig. 2b), whereas NLR, PLR, and CRP were not significant for alcoholic AP (NLR-AUC 0.618, 95% CI 0.51–0.72; PLR-AUC 0.446, 95% CI 0.34–0.55; CRP-AUC 0.598, 95% CI 0.49–0.71, Fig. 2c). The best NLR and PLR cutoffs for predicting POF in gallstone AP patients were 7.8 and 229.1, respectively, with sensitivity and specificity of 88.9% and 41.1% for NLR and 70.4% and 52.3% for PLR.

## Discussion

In the present study, we investigated the value of NLR and PLR as predictive markers of AP severity. We found that NLR and PLR were well correlated with other scoring systems in patients with gallstone AP. In addition, NLR and PLR showed significant predictive ability for POF in patients with gallstone AP. However, in patients with alcoholic AP, NLR and PLR were not correlated with other scoring systems.

NLR was first introduced as an easily measurable parameter assessing systemic inflammation and stress in critically ill patients [11]. Later, PLR was also found to be an inflammatory marker, and the role of platelets as a critical link between inflammation and microvascular dysfunction has since been investigated [12–14]. The prognostic value of these two parameters has been confirmed in a variety of clinical conditions, and PLR was shown to be superior to NLR in certain cancers [8–10]. AP is an inflammatory condition characterized by activation of both innate and adaptive immune responses. Activation and modulation of neutrophils and platelets play a core role in establishing host defenses in settings of systemic inflammation; however, excessive inflammatory response causes massive cell transmigration to the pancreas and subsequent release of aggressive defense molecules, resulting in destruction of the pancreas and organ failure [15–18].

A few studies have investigated the relationship between NLR and outcome in patients with AP. Azab et al. [7] first applied the concept of NLR to patients with AP in 2011. They found that NLR was a better predictor of ICU admission or prolonged hospitalization in AP than was total WBC count and suggested a cutoff value of < 4.7 as a predictor of a poor outcome [7]. However, in-hospital mortality was extremely low in that study, and the investigators failed to assess records of organ failure. Suppiah et al. [6] revealed an association between NLR measured in the first 48 h and the risk of AP developing into a more severe form. However, that study was limited by a small sample size (*n* = 146), and the AP cases included were mostly mild, with no local/systemic complications or organ failure. Recently, Gulen et al. [19] investigated the association between NLR and early mortality and argued that NLR is not a significant independent prognostic factor. Zhang et al. [2] demonstrated that elevated NLR is associated with POF, ICU stay longer than > 7 days, and increased in-hospital mortality in a Chinese population.

However, despite the demonstrated superiority of PLR over NLR in predicting the outcome of inflammation in several clinical conditions, no previous study has investigated the predictive value of PLR at the time of admission on outcomes in patients with AP. Furthermore, although they are grouped together as pancreatitis, gallstone AP and alcoholic AP each has a distinct pathophysiology, and no study has compared NLR or PLR between them. Therefore, we investigated the value of PLR in predicting AP outcomes and compared differences between NLR and PLR patterns in two distinct forms of AP. We excluded pancreatitis caused by factors other than gallstone or alcohol. When all AP cases were combined, NLR and PLR were not significant independent predictive factors of POF. However, after subgrouping AP by etiology, both NLR and PLR were independent predictive factors of POF in gallstone AP. In alcoholic AP, NLR was a significant predictor, but PLR was not. This can be explained by the different mechanism of alcohol AP. Alcoholic AP is usually associated with chronic liver disease. In our results, the number of liver cirrhosis patients was larger and the platelet count was lower in alcoholic AP compared to gallstone AP. Thrombocytopenia is related to chronic liver disease due to impaired platelet production and decreased hepatic synthesis of thrombopoietin [20]. Therefore, PLR can vary according to liver function as well as systemic inflammation. Interestingly, CRP, a marker traditionally used to assess the severity of inflammation [21–23], failed to predict POF in gallstone AP and alcoholic AP. This suggests the superiority of NLR and PLR to CRP in predicting the course of gallstone AP.

Although the exact mechanism of alcoholic AP has not been elucidated, our findings imply a fundamental difference in pathophysiology between the two subgroups. Although elevated NLR and PLR can be used as predictive biomarkers in AP, interpretation should follow confirmation of the etiology. Furthermore, our findings challenge the rationale of applying a uniform prognostic scoring system to all AP. Replacing WBC count with NLR or PLR in traditional prognostic scoring systems could improve their performance [10].

Also, pancreatic cancer can induce impairment of the patient’s immune system through systemic inflammation [24]. In this aspect, NLR and PLR can reflect the status of the immune system in patients with pancreatic cancer. Several studies have reported that NLR and PLR were correlated with poor overall survival in patients with pancreatic cancer [25, 26]. A recent study demonstrated that NLR was a predictive marker for the presence of invasive carcinoma in patients with intraductal papillary mucinous neoplasm [27]. Therefore, NLR and PLR have a role as biomarkers in pancreatic malignancy.

This study has several limitations. First, the number of patients enrolled in this study was small, and this study was performed in a tertiary care center, which could have resulted in disproportional inclusion of patients with severe disease status and tendency to progress to POF. Such selection bias might have overestimated the predictive value of elevated NLR or PLR. Second, we did not compare NLR or PLR with other biochemical markers, such as procalcitonin and IL-6. Third, we did not describe changes in NLR or PLR during treatment, which could estimate the prognosis of AP. Despite these limitations, this study also has strengths. This is the first prospective study investigating the predictive value of PLR in AP and the difference between NLR and PLR in two subgroups of AP. Also, all laboratory values were obtained within 1 h of initial presentation, minimizing changes in WBC and platelet counts caused by hydration and medication.

## Conclusions

In conclusion, both NLR and PLR were significant independent predictive factors of POF in gallstone AP, and they were better predictors of POF than was CRP, a traditionally used inflammatory marker and independent prognostic factor. Future studies including a larger number of patients across both subgroups of AP should be performed to further compare differences between NLR and PLR in the two etiologies.

## Abbreviations

AP: Acute pancreatitis
APACHE II: Acute Physiologic Assessment and Chronic Health Evaluation II
AUC: Area under the curve
BISAP: Bedside Index for Severity in Acute Pancreatitis
CRP: C-reactive protein
CTSI: Computed tomography scoring index
NLR: Neutrophil to lymphocyte ratio
PLR: Platelet to lymphocyte ratio
POF: Persistent organ failure
SIRS: Systemic inflammatory response syndrome
WBC: Total white blood cell
